# Sex‑specific associations between multiple hormone axes and dynapenic obesity in middle‑aged and older patients with type 2 diabetes: A cross-sectional study

**DOI:** 10.1097/MD.0000000000050242

**Published:** 2026-08-28

**Authors:** Di Yu, Qin Ou, Bo Xie, Yinghao Li, Kun Liao, Rong Li, Mei Mei, Bo Zhou

**Affiliations:** a Department of Endocrinology, The First Affiliated Hospital of Chongqing Medical University, Chongqing, China; b Department of Endocrinology, The Affiliated Dazu’s Hospital of Chongqing Medical University, The People’s Hospital of Dazu, Chongqing, China; c Department of Cardiovascular Medicine, Zhuzhou Central Hospital, Zhuzhou Hospital Affiliated to Xiangya School of Medicine, Central South University, Zhuzhou, Hunan, China.

**Keywords:** dynapenic obesity, hormones, sarcopenic obesity, type 2 diabetes

## Abstract

Accumulating evidence indicates that hormones play a critical role in the pathogenesis of dynapenic obesity (DO), although the precise relationships and underlying mechanisms remain elusive. This study aimed to investigate sex-stratified associations between multiple hormones and prevalence of DO in type 2 diabetes mellitus (T2DM) populations. In this cross-sectional study of 557 T2DM patients, body composition was assessed by dual-energy X-ray absorptiometry, and hormones from pituitary-target gland axes and the vitamin D system were also tested. Moreover, demographic data, nutritional and inflammatory status, comorbidities and diabetic complications were collected. On the basis of sex-specific cutoff values for grip strength and body fat percentage, the participants were categorized into 4 groups: nondynapenic/nonobese (ND/NO), nondynapenic/obese (ND/O), dynapenic/nonobese (D/NO), and DO. Ordinal logistic regression was used to analyze the associations between the progression of low muscle strength and concurrent adipose accumulation and hormonal profiles. Among the enrolled diabetic adults, the incidence rates of DO in males and females were 20.6% and 37.9%, respectively. Compared with ND/NO patients, DO patients typically exhibit advanced age, poorer nutritional status, reduced gait speed and low-grade inflammation. Moreover, the 25 (OH) D level in males with DO was significantly lower than that in the ND/NO group, and even after full adjustment, 25 (OH)D remained significantly inversely associated with DO progression (OR = 0.98, 95% CI: 0.96–1.00, *P* < .05); however, in females, the serum dehydroepiandrosterone level also decreased (OR = 0.99, 95% CI: 0.99–1.00, *P* < .05), whereas the parathyroid hormone level increased (OR = 1.01, 95% CI: 1.00–1.02, *P* < .05) with increasing DO. DO is relatively common in individuals with T2DM and is closely related to aging, nutritional and inflammatory status, and sex-specific hormonal differences.

## 1. Introduction

With changing lifestyles and extended lifespan, the significance of sarcopenic obesity (SO) in individuals with type 2 diabetes mellitus (T2DM) has become increasingly recognized. A meta-analysis revealed that the global prevalence of SO has reached as high as 11%^[1]^ but varies greatly across subsets of populations, with an elevated prevalence of 34.5% in individuals with T2DM (12.7% in non-T2DM populations).^[2]^ On the other hand, another meta-analysis revealed that SO patients have a 38% increased risk of developing T2DM (OR: 1.38, 95% CI: 1.27–1.50) compared with their non-SO counterparts. These findings suggest that there is a complex interplay between diabetes and SO. More unfortunately, a growing body of evidence has suggested that, compared with T2DM alone, the coexistence of SO and T2DM can significantly exacerbate the risk of cognitive decline, insulin resistance, adverse cardiovascular outcomes, and even all-cause mortality.^[3–7]^ Therefore, elucidating the pathological mechanisms underlying SO in T2DM patients and developing effective interventions holds substantial clinical importance.

However, the reason SO is more common in patients with T2DM is still incompletely understood; multiple factors, such as aging, dietary changes, sedentary behavior, insulin resistance, low-grade inflammation, and potential alterations in hormonal profiles, may be involved.^[8]^ Among them, hormones, as systemic and modifiable bioactive molecules, have long been a focus of research. However, to date, only a limited number of studies have preliminarily explored hormonal characteristics in patients with SO. For example, a cross-sectional study revealed that insulin-like growth factor-1 (IGF-1) concentrations may be strongly negatively associated with the occurrence of SO.^[9]^ Another study suggested that postmenopausal women frequently experience fat accumulation and a decrease in lean mass, potentially linked to a decrease in estrogen levels.^[10]^ Similarly, a study from the New Mexico Elder Health Survey revealed that male participants with SO presented significantly lower testosterone and IGF-1 levels than non-SO controls did.^[11]^ Additionally, reduced 25 (OH)D has recently been linked to the incidence of SO.^[12]^ However, the above studies predominantly examined the isolated hormone axis in the context of SO and rarely included T2DM patients.

SO is characterized by a decrease in muscle mass and function in obese individuals, but consensus-based diagnostic cutoffs for lean body mass adjusted by weight remain undefined in Asian populations. Dynapenic obesity (DO), a clinical subtype of SO, is more readily identifiable and diagnostically accessible. Nevertheless, a large gap still exists in the understanding of the phenotypic and hormonal profiles of such patients, particularly those complicated by T2DM. Thus, this study aimed to investigate biological sex-specific phenotypic features and multihormonal dynamics in DO patients with T2DM, with the ultimate goal of identifying potential targets for hormonal modulation and therapy.

## 2. Materials and methods

### 2.1. Study design and participants

**Figure 1.**
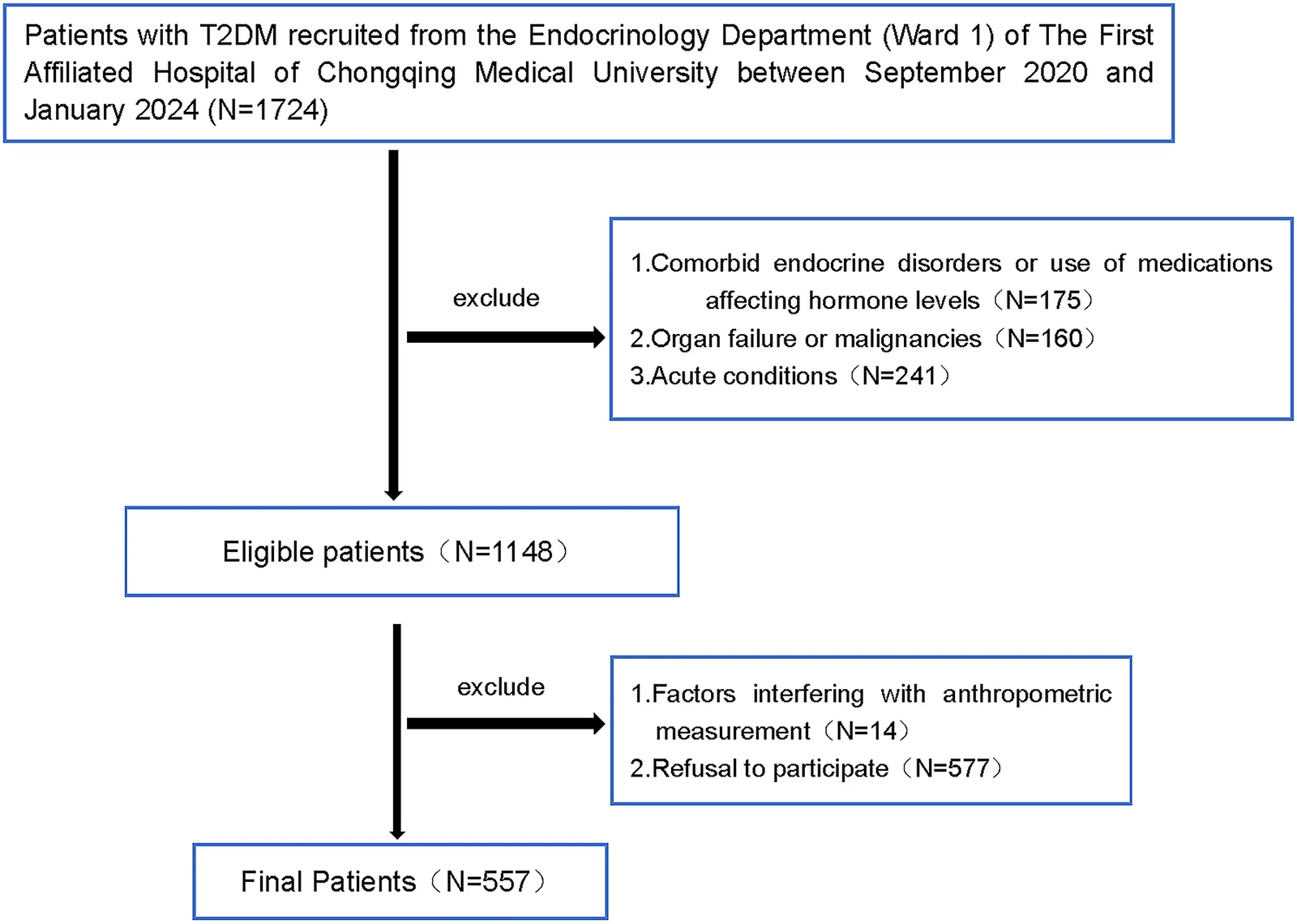
Study flow chart of the participants.

This was a cross-sectional study in which patients were consecutively recruited from the Department of Endocrinology of the First Affiliated Hospital of Chongqing Medical University between September 2020 and January 2024. Eligible individuals were aged 40 years or older with T2DM according to the World Health Organization 1999 criteria. As shown in Figure 1, a total of 1724 patients with T2DM were included during the study period. The exclusion criteria were as follows: comorbid endocrine disorders or the use of medications affecting hormone levels, such as thyroid dysfunction; hyperparathyroidism; endogenous or exogenous hypercortisolism; or sex hormone or thyroid hormone replacement; organ failure or malignancies, such as heart failure, cirrhosis, renal failure, chronic pulmonary disease, breast cancer, lung cancer or prostate cancer; acute conditions, such as infection, trauma, pregnancy, or hyperglycemic crises; factors interfering with anthropometric measurements, such as edema, amputation, limb injury, or implants; and refusal to participate. Ultimately, 557 eligible participants were included in the current analysis. Owing to the retrospective design, the requirement for written informed consent from participants was waived.

### 2.2. Anthropometric assessment and grouping

Trained technicians assessed body composition and areal bone mineral density (aBMD) using dual-energy X-ray absorptiometry Discovery A, S/N 87352, Hologic, Bedford, USA). The parameters included body fat percentage (BF%), visceral fat area (VFA), appendicular lean mass, and aBMD at the lumbar spine, femoral neck, and total hip. Grip strength was measured by 2 technicians using standardized protocols: grip strength was assessed twice with an electronic dynamometer (EH101, CAMRY, Guangdong, China) on the dominant hand, with the highest value recorded. In addition, gait speed was determined by timing a 6-meter walk at usual pace. Waist circumference was measured at the midpoint between the lower rib margin and the iliac crest.

On the basis of the Asian Working Group for Sarcopenia criteria established in 2019, low muscle strength was defined as handgrip strength <28 kg in males and <18 kg in females.^[13]^ Obesity was defined as meeting one of 3 different criteria: body mass index (BMI) ≥ 25 kg/m^2^ (World Health Organization Western Pacific Region guidelines), BF% >25% (men) or >30% (women) (National Health Commission of China), or VFA ≥ 100 cm^2^ (Chinese Medical Association guidelines). DO was defined as obesity combined with low handgrip strength. The participants were categorized into 4 groups: nondynapenic/nonobese (ND/NO), nondynapenic/obese (ND/O), dynapenic/nonobese (D/NO), and DO.

### 2.3. Hormone assays

Chemiluminescence immunoassays and kits were used to measure adrenocorticotropic hormone (Shenzhen, China) with a Mindray 6000i analyzer and 25 (OH)D (DiaSorin, Italy) with a LiaisonXL analyzer. Electrochemiluminescence was used to measure IGF-1 (Elecsys, Roche Diagnostics GmbH, Mannheim, Germany). The levels of sex hormones, including estradiol, progesterone, testosterone (T), prolactin, follicle-stimulating hormone, luteinizing hormone, sex hormone-binding globulin (SHBG), dehydroepiandrosterone (DHEAS), parathyroid hormone (PTH), cortisol, and thyroid function markers (free triiodothyronine (FT3), free thyroxine (FT4), and thyroid-stimulating hormone (TSH)), were measured via chemiluminescence with kits (Beckman Coulter) on a DXI800 analyzer. The free estrogen index and free androgen index are both obtained through calculation.

### 2.4. Data collection

Demographics (age, sex, smoking, alcohol use, BMI, diabetes duration), as well as diabetic complications (retinopathy, nephropathy, neuropathy) and comorbidities (hypertension, coronary heart disease, stroke, falls, fractures), were extracted from electronic records. Diabetes severity was evaluated using the diabetes complications severity index. In addition, metabolic parameters (fasting plasma glucose, glycated hemoglobin (HbA1c), homeostatic model assessment of insulin resistance (HOMA-IR), lipids, calcium, phosphorus, estimated glomerular filtration rate (eGFR)) and nutritional-inflammatory markers (hemoglobin, albumin, high-sensitivity C-reactive protein (hsCRP)) were also obtained from medical records.

### 2.5. Definitions and calculations

DCSI: Scored as 0 (none), 1 (mild), or 2 (severe) across 7 complications (retinopathy, nephropathy, neuropathy, peripheral artery disease, metabolic crises, cardiovascular, and cerebrovascular disease).^[14]^ eGFR was calculated according to the Chronic Kidney Disease Epidemiology Collaboration (CKD-EPI) formula. HOMA-IR: 1.5 + (fasting glucose × C-peptide)/2800.^[15]^ Skeletal muscle index: appendicular lean mass/height^2^. FEI: 100 × (total estradiol × 0.003671)/SHBG.^[16]^ Free androgen index: 100 × (total testosterone × 3.467)/SHBG.^[17]^ BMI: Weight/height^2^. Adjusted [Ca] (mmol/L) = total [Ca] (mmol/L) + 0.02 (40–[albumin] (g/L)).^[18]^

### 2.6. Statistical analysis

The data were analyzed using SPSS 29.0. Normality was assessed via the Shapiro–Wilk test. Continuous variables are presented as medians with interquartile ranges (25%–75%), or means ± standard deviations, and categorical variables are presented as frequencies and percentages. One-way ANOVA was used for group comparisons, and the least significant difference test was used for post hoc comparisons (normal data). Nonnormally distributed variables were compared by the Kruskal–Wallis test. The chi-square test or Fisher’s exact test was used to compare categorical variables. We have performed the Test of Parallel Lines in SPSS. The result showed no statistical significance (*P* > .05). We then assigned 2 to the ND/NO group, 1 to the D/NO and ND/O groups, and finally assigned 0 to the DO group to examine the relationships between changes in muscle strength and fat mass and hormone levels across 3 different models using OLR. Model 1 was unadjusted, Model 2 was adjusted for demographic factors (age, smoking status and alcohol use), and Model 3 was additionally adjusted for diabetes-related factors (HbA1c and DCSI scores).

## 3. Results

### 3.1. Diagnostic performance of the DO

**Figure 2.**
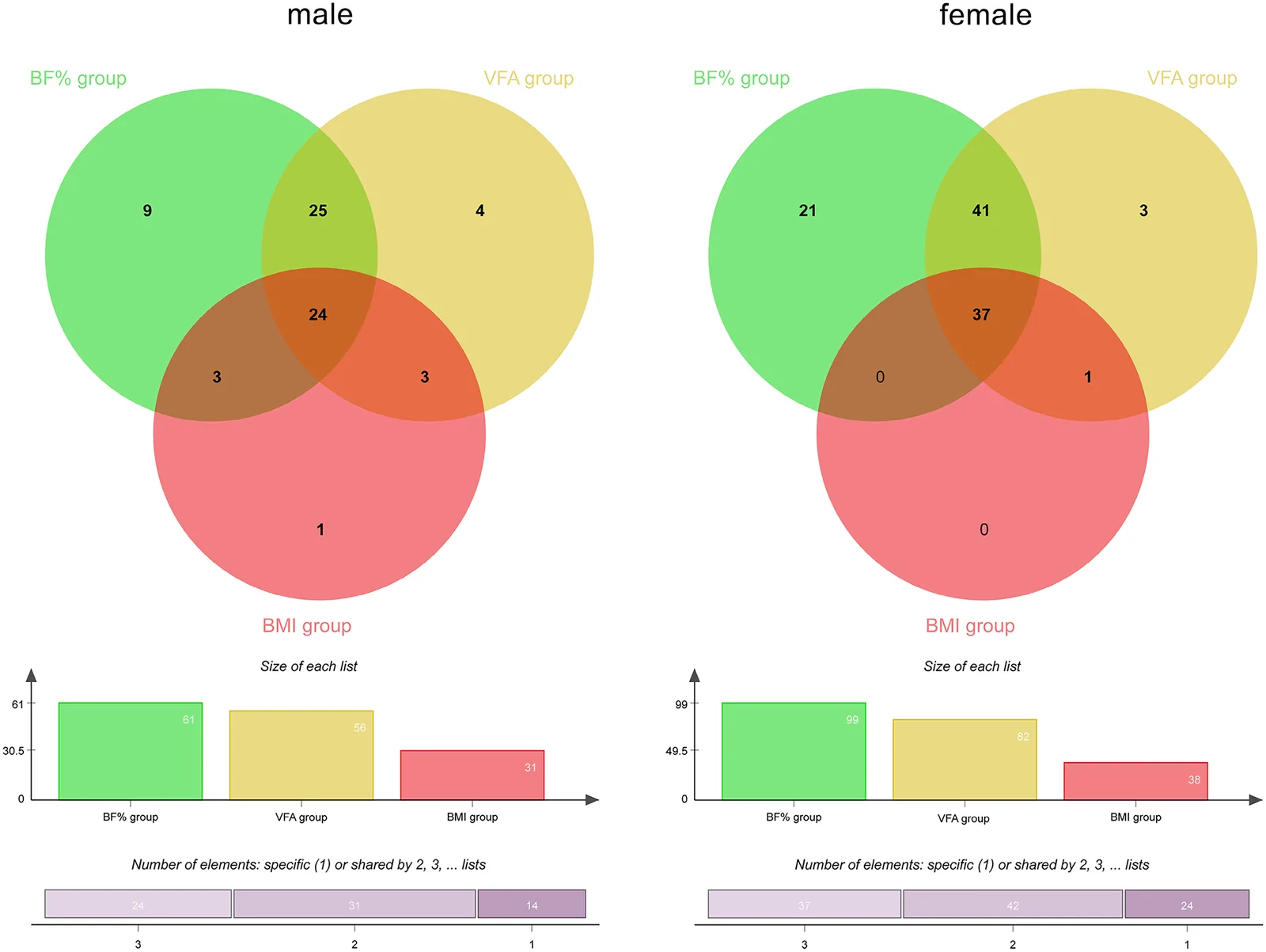
Number of participants classified as having dynapenic obesity in males (left) and females (right) according to each definition. The green circle represents dynapenic obesity defined by BF%, the yellow circle represents dynapenic obesity defined by VFA, and the red circle represents dynapenic obesity defined by BMI. BF% = body fat percentage, VFA = visceral fat area, BMI = body mass index.

The DO prevalence varies with the definition of obesity, despite the same criteria for low muscle strength being adopted. Figure 2 shows the rate of overlap and the number of participants classified as DO according to each definition. When obesity was defined according to BF%, VFA, and BMI, the number of DO cases in males accounted for 61 cases (20.6%), 56 cases (18.9%), and 31 cases (10.4%), respectively, whereas in females, it accounted for 99 cases (37.9%), 82 cases (31.4%), and 38 cases (14.5%), respectively. In addition, further analysis suggested that the number of people identified with DO on the basis of BMI was much lower than that identified with the other 2 methods. Notably, among males, only 24 patients (8.1%) with DO met all 3 criteria; likewise, only 37 patients (14.2%) among females met all 3 criteria. Considering that the European Society of Clinical Nutrition and Metabolism (ESPEN) and the European Association for the Study of Obesity (EASO) have explicitly proposed the use of BF% to identify SO in 2022,^[19]^ this study ultimately also used BF% as one of the diagnostic criteria for DO.

### 3.2. Baseline characteristics

Among the 557 participants included in this study, 296 were males (53.1%), and 261 were females (46.9%). Table 1 shows the baseline characteristics of the male participants in each group. 61 (20.6%) patients were in the DO group, 24 (8.1%) patients were in the D/NO group, 141 (47.9%) patients were in the ND/O group, and 69 (23.3%)patients were in the ND/NO group. Compared with those with ND/NO (control group), individuals with DO tended to be older, and the eGFRs were significantly lower. Although fasting C-peptide and the inflammatory marker hsCRP seemed to be higher, and fasting plasma glucose, total cholesterol, hemoglobin and albumin were lower in the DO group than in the control group, there were no significant differences between the DO group and the control group. Similarly, there were no differences in smoking, drinking status, comorbidities or diabetic complications between DO patients and those in the control group.

**Table 1:** The baseline characteristics of males in each group.

	Dynapenic obesity n = 61 (20.6%)	Dynapenic nonobesity n = 24 (8.1%)	Nondynapenic obesity n = 142 (47.9%)	Nondynapenic nonobesity n = 69 (23.3%)	*P*
Demographics					
Age (yr)	68.05 (62.00, 76.00)‡^,^§	67.99 (64.01, 76.25)‡^,^§	63.00 (56.00, 70.00)*^,^†	60.00 (55.00, 65.53)*^,^†	<.001
Smoke (%)	35 (57.4%)	16 (66.7%)	80 (56.7%)	48 (69.6%)	.279
Drink (%)	21 (34.4%)	6 (25%)	59 (41.8%)	27 (39.1%)	.399
Diabetes duration (yr)	14.00 (9.00, 20.00)	14.00 (10.00, 19.00)	10.00 (2.00, 18.00)	10.00 (7.00, 15.00)	.090
Metabolic parameters					
Fasting plasma glucose(mmol/L)	7.60 (6.80, 10.00)	7.75 (6.58, 10.05)	8.00 (5.90, 10.70)§	9.20 (7.70, 11.60)‡	.044
Glycosylated hemoglobin (%)	9.20 (7.20, 11.20)	9.50 (8.45, 12.18)	8.90 (7.60, 10.80)	9.00 (7.00, 10.35)	.149
Fasting C-peptide (ng/mL)	2.27 (1.31, 3.80)	1.37 (0.85, 2.20)	1.92 (1.31, 3.05)	1.69 (0.91, 3.04)	.043
HOMA-IR	1.51 (1.50, 1.51)†	1.50 (1.50, 1.51)*	1.51 (1.50, 1.51)	1.51 (1.50, 1.51)	.059
Total cholesterol (mmol/L)	4.10 (3.23, 4.74)	4.38 (3.59, 4.96)	3.90 (3.23, 4.76)	4.38 (3.68, 5.34)	.039
Triglycerides (mmol/L)	1.42 (0.92, 2.06)	1.07 (0.90, 1.48)	1.38 (0.99, 2.09)	1.34 (0.76, 2.26)	.455
HDL-C (mmol/L)	1.06 (0.89, 1.25)	1.10 (0.93, 1.31)	0.99 (0.87, 1.17)	1.04 (0.92, 1.26)	.096
LDL-C (mmol/L)	2.38 (1.61, 3.16)	2.76 (2.07, 3.29)	2.30 (1.73, 2.95)	2.58 (2.06, 3.17)	.257
Creatiinine (umol/L)	86.00 (66.00, 102.00)	82.50 (64.50, 109.25)	75.00 (68.00, 89.00)	76.00 (64.00, 88.50)	.195
Uric acid (umol/l)	343.00 (286.00, 387.00)	314.50 (264.50, 392.25)	335.00 (283.00, 384.00)	311.00 (252.50, 375.00)	.263
eGFR (ml/min/1.73m^2^)	114.65 (93.28, 142.13)‡^,^§	120.61 (85.87, 162.38)‡^,^§	135.28 (110.00, 155.05)*^,^†	133.14 (111.51, 163.88)*^,^†	.011
Albumin-adjusted-Calcium (mmol/L)	2.29 (2.21, 2.37)	2.33 (2.22, 2.41)	2.27 (2.19, 2.35)	2.24 (2.16, 2.37)	.369
Phosphorus (mmol/L)	1.20 (1.08, 1.30)	1.21 (0.98, 1.37)	1.18 (1.08, 1.31)	1.23 (1.03, 1.34)	.922
Magnesium (mmol/L)	0.83 (0.76, 0.89)	0.84 (0.75, 0.89)	0.82 (0.76, 0.87)	0.83 (0.75, 0.88)	.790
Nutritional and inflammatory markers					
Hemoglobin (g/L)	139.00 (124.00, 145.00)‡	130.00 (110.50, 146.50)‡	146.00 (138.00, 154.00)*^,^†	144.00 (132.50, 155.00)	<.001
Albumin (g/L)	41.00 (39.00, 44.00)	39.00 (36.00, 43.75)‡	42.00 (39.00, 44.00)†	42.00 (39.00, 44.00)	.026
hsCRP (mg/L)	1.39 (0.49, 3.38)	0.63 (0.30, 1.80)	0.97 (0.49, 3.09)	0.70 (0.38, 2.31)	.171
Comorbidities					
Stroke (%)	7 (11.5%)	3 (12.5%)	11 (7.8%)	6 (8.7%)	.790
Hypertension (%)	40 (65.6%)§	13 (54.2%)	83 (58.9%)§	30 (43.5%)*^,^‡	.067
Coronary heart disease(%)	16 (26.2%)	4 (16.7%)	29 (20.6%)	10 (14.5%)	.394
Fracture (%)	4 (6.6%)	3 (12.5%)	4 (2.8%)	3 (4.3%)	.132
Fall (%)	3 (4.9%)	1 (4.2%)	1 (0.7%)	2 (2.9%)	.160
Diabetic complication					
Kidney disease (%)	21 (34.4%)†	14 (58.3%)*^,^‡^,^§	42 (29.8%)†	23 (33.3%)†	.058
Peripheral neuropathy (%)	42 (68.9%)‡	18 (75%)‡	74 (52.5%)*^,^†	40 (58%)	.058
Retinopathy (%)	19 (31.1%)	12 (50%)‡	38 (27%)†	24 (34.8)	.138
DCSI score	2.00 (1.00, 3.00)	3.00 (2.00, 4.00)‡	2.00 (1.00, 3.00)†	2.00 (1.00, 3.00)	.029

DSCI = diabetes complications severity index, eGFR = estimated glomerular filtration ratio, HDL-C = high-density lipoprotein-cholesterol, HOMA-IR = homeostatic model assessment of insulin resistance, hsCRP = hypersensitive C-reactive protein, LDL-C = low-density lipoprotein-cholesterol.

* Significantly different from dynapenic obesity group.

† Significantly different from dynapenic nonobesity group.

‡ Significantly different from nondynapenic obesity group.

§ Significantly different from nondynapenic nonobesity group. (The same annotations apply to subsequent tables).

Among females, 99 patients (37.9%) were in the DO group, 17 patients (6.5%) were in the D/NO group, 123 patients (47.1%) were in the ND/O group, and 22 patients (8.4%) were in the ND/NO group. As shown in Table 2, participants in the DO group were also older and had higher hsCRP and lower levels of hemoglobin than those in the reference group. Other metabolic parameters, such as albumin-corrected calcium, fasting plasma glucose and HbA1c, were lower, but there were no significant differences between the DO group and the control group. Similarly, there were no differences in clinical comorbidities or diabetes complications between DO patients and control patients.

**Table 2:** The main baseline characteristics of female in each group

	Dynapenic obesity n = 99 (37.9%)	Dynapenic nonobesity n = 17 (6.5%)	Nondynapenic obesity n = 123 (47.1%)	Nondynapenic nonobesity n = 22 (8.4%)	*P*
Demographics					
Age (yr)	70.00 (64.00, 75.30)‡^,^§	60.88 (56.50, 72.44)	67.00 (59.50, 73.00)*	60.00 (55.00, 70.00)*	<.001
Smoke (%)	3 (3.0%)	1 (5.9%)	6 (4.8%)	4 (14.8%)	.160
Drink (%)	23 (23.2%)	1 (5.9%)	33 (26.2%)	8 (29.6%)	.352
Diabetes duration (yr)	10 (6.00, 20.00)	10 (2.00, 20.00)	10 (3.00, 20.00)	10 (2.00, 20.00)	.196
Metabolic parameters					
Fasting plasma glucose (mmol/L)	7.20 (5.70, 9.00)†	9.50 (7.25, 14.30)*	7.60 (6.00, 11.00)	7.70 (5.60, 13.20)	.018
Glycosylated hemoglobin (%)	7.65 (6.30, 9.55)	9.80 (6.45, 11.95)	8.90 (6.70, 10.50)	8.70 (6.80, 11.80)	.027
Fasting c-peptide (ng/mL)	2.35 (1.38, 3.77)	1.67 (0.95, 3.86)	2.28 (1.29, 6.47)	1.42 (0.73, 3.24)	.156
HOMA-IR	1.51 (1.50, 1.52)	1.51 (1.50, 1.52)	1.51 (1.50, 1.52)	1.51 (1.50, 1.52)	.932
Total cholesterol (mmol/L)	4.19 (3.34, 5.14)	4.10 (3.32, 4.50)	4.19 (3.44, 5.16)	4.62 (3.84, 5.49)	.097
Triglycerides (mmol/L)	1.32 (0.94, 1.96)	1.01 (0.78, 1.79)	1.34 (0.92, 2.06)	1.58 (0.71, 3.15)	.746
HDL-C (mmol/L)	1.19 (1.01, 1.42)	1.20 (0.72, 1.49)	1.16 (0.95, 1.38)	1.21 (1.00, 1.38)	.612
LDL-C (mmol/L)	2.36 (1.56, 3.03)	2.27 (2.12, 3.49)	2.49 (1.86, 3.16)	2.72 (1.84, 3.35)	.435
Creatiinine (umol/L)	63.00 (50.00, 85.00)	62.00 (51.50, 81.00)	60.00 (51.00, 71.00)	72.00 (56.00, 85.00)	.088
Uric acid (umol/L)	288.00 (241.00, 376.25)	271.00 (209.50, 389.65)	289.00 (225.50, 378.95)	310.00 (266.00, 370.00)	.813
eGFR (ml/min/1.73m^2^)	127.10 (94.28, 132.00)	127.46 (96.21, 132.27)	128.02 (111.45, 132.00)	128.60 (89.27, 132.00)	.496
Albumin-adjusted-Calcium (mmol/L)	2.25 (2.19, 2.33)‡	2.27 (2.21, 2.33)	2.28 (2.24, 2.37)*	2.27 (2.19, 2.35)	.031
Phosphorus (mmol/L)	1.24 (1.08, 1.36)	1.29 (1.23, 1.43)	1.25 (1.16, 1.36)	1.25 (1.15, 1.46)	.312
Magnesium (mmol/L)	0.82 (0.77, 0.88)	0.82 (0.76, 0.84)	0.82 (0.77, 0.86)	0.82 (0.77, 0.87)	.896
Nutritional and inflammatory markers					
Hemoglobin (g/L)	124.00 (116.75, 135.00)‡^,^§	117.00 (105.50, 130.50)‡^,^§	131.00 (124.00, 141.00)*^,^†	136.00 (123.00, 144.00)*^,^†	<.001
Albumin (g/L)	41.00 (38.00, 44.00)	40.00 (34.50, 45.00)	41.00 (38.00, 44.00)	42.00 (39.00, 45.00)	.427
hsCRP (mg/l)	1.22 (0.51, 3.43)§	1.92 (0.54, 5.79)	1.03 (0.28, 943)	0.61 (0.31, 1.88)*	.019
Combidities					
Stroke(%)	5 (5.1%)	0 (0.00)	9 (7.1%)	0 (0.00)	.557
Hypertension (%)	63 (63.6%) †	5 (29.4%) *^,^‡	69 (54.8%) †	16 (59.3%)	.055
Coronary heart disease(%)	24 (24.2%) †	0 (0.00)*	23 (18.3%)	3 (11.1%)	.057
Fracture(%)	15 (15.3%)	3 (17.6%)	26 (21.0%) §	0 (0.00)‡	.060
Fall (%)	8 (8.2%)	2 (11.8%)	11 (8.9%)	0 (0.00)	.460
Diabetic complications					
Kidney disease (%)	32 (32.7%)	5 (29.4%)	33 (26.6%)	7 (30.4%)	.820
Peripheral neuropathy (%)	62 (63.3%)	13 (76.5%)	67 (54.0%)	11 (47.8%)	.150
Retinopathy (%)	35 (35.7%) ‡	9 (52.9%) ‡	28 (22.6%) *^,^†^,^§	11 (47.8%) ‡	.010
DCSI score	0.28 (0.25, 0.30)	2.00 (1.00, 3.00)	2.00 (1.00, 3.00)	2.00 (1.00, 3.00)	.304

The significance value for the footnotes “*, †, ‡, §” is *P* < .05.

With respect to the anthropometric parameters across different biological sexes, consistent results were obtained, as shown in Table 3. Regardless of sex, the waist circumference, BF%, and VFA of the DO group were significantly greater than those of the control group, whereas the ASM/W, gait speed and muscle strength were lower. However, there was no significant difference in aBMD between the DO group and the other groups.

**Table 3:** The muscle function and anthropometry of different sexes in each group.

	Dynapenic obesity	Dynapenic nonobesity	Nondynapenic obesity	Nondynapenic nonobesity	*P*
Male					
Body parameters					
Waist circumference (cm)	92.00 (89.00, 97.00)†^,^§	84.75 (79.13, 92.00)*^,^‡	92.00 (87.50, 98.50)†^,^§	88.00 (84.50, 92.00)*^,^‡	<.001
Body fat percentage (%)	28.10 (26.20, 31.50)†^,^§	23.35 (20.53, 23.98)*^,^‡	27.30 (26.00, 29.60)†^,^§	22.20 (20.80, 24.00)*^,^‡	<.001
Visceral fat area (cm^2^)	126.00 (105.00, 159.00)†^,^§	80.55 (58.35, 103.70)*^,^‡	134.00 (115.00, 157.00)†^,^§	89.60 (76.70, 105.50)*^,^‡	<.001
Body mass index (kg/m^2^)	24.03 (22.57, 26.61)†	21.84 (19.96, 23.80)*^,^‡	24.89 (23.63, 26.81)†^,^§	23.05 (21.53, 24.99)‡	<.001
ASMI (kg/m^2^)	6.47 (6.00, 7.11)‡^,^§	6.66 (6.08, 7.16)‡	7.21 (6.83, 7.74)*^,^†	7.03 (6.46, 7.62)*	<.001
ASM/W	0.27 (0.26, 0.28)†^,^‡^,^§	0.31 (0.29, 0.33)*^,^‡	0.29 (0.28, 0.30)†^,^§	0.31 (0.29, 0.32)*^,^‡	<.001
Grip strength (kg)	21.60 (19.80, 25.90)‡^,^§	24.20 (21.70, 26.40)‡^,^§	35.40 (31.30, 38.80)*^,^†	36.20 (32.80, 38.75)*^,^†	<.001
Gait speed (m/s)	0.92 (0.77, 1.14)‡^,^§	0.82 (0.54, 0.97)‡^,^§	1.12 (0.90, 1.29)*^,^†	1.06 (0.90, 1.28)*^,^†	<.001
aBMD					
Lumbar spine T-Scores	−1.53 (±1.34)	−1.80 (±1.71)	−1.29 (±1.21)	−1.48 (±1.23)	0.262
Femoral neck T-Scores	−1.70 (−2.30,−1.20)	−1.75 (−2.60,−1.33)	−1.50 (−1.90,−1.10)	−1.54 (−1.90,−1.00)	0.034
Total hip T-Scores	−1.10 (−1.60,−0.80)	−1.20 (−2.08,−0.85)	−1.10 (−1.40,−0.40)	−1.10 (−1.60,−0.55)	0.159
Female					
Body parameters					
Waist circumference (cm)	88.25 (78.50, 92.25)†^,^§	74.00 (68.75, 86.75)*^,^‡	89.00 (81.00, 92.75)†^,^§	80.50 (67.00, 88.50)*^,^‡	<.001
Body fat percentage (%)	35.70 (33.15, 39.00)†^,^§	28.70 (24.95, 29.65)*^,^‡	35.60 (33.15, 38.70)†^,^§	27.40 (24.50, 28.40)*^,^‡	<.001
Visceral fat area (cm^2^)	127.70 (103.75, 153.75)†^.^§	58.10 (45.95, 90.95)*^,^‡	125.00 (98.85, 152.65)†^,^§	84.30 (53.00, 124.00)*^,^‡	<.001
Body mass index (kg/m^2^)	23.83 (22.26, 26.04)†^,^§	19.65 (19.11, 21.22)*^,^‡	24.38 (22.82, 26.48)†^,^§	22.60 (20.31, 24.03)*^,^‡	<.001
ASMI (kg/m^2^)	5.57 (±0.70)‡	5.25 (±0.45)‡^,^§	5.88 (±0.69)*^,^†	6.11 (±0.92)†	<.001
ASM/W	0.23 (±0.02)†^,^§	0.26 (±0.02)*^,^‡	0.24 (±0.02)†^,^§	0.28 (±0.03)*^,^‡	<.001
Grip strength (kg)	14.70 (11.60, 16.40)‡^,^§	14.40 (12.05, 15.60)‡^,^§	21.30 (19.75, 23.25)*^,^†^,^§	28.40 (22.20, 33.20)*^,^†^,^‡	<.001
Gait speed (m/s)	0.81 (0.66, 0.93)‡^,^§	0.93 (0.68, 1.04)	0.94 (0.81, 1.08)	1.19 (0.94, 1.38)†^,^‡	<.001
aBMD					
Lumbar spine T-Scores	−2.40 (±1.16)	−2.62 (±0.99)	−2.32 (±1.18)	−2.03 (±0.92)	.344
Femoral neck T-Scores	−2.16 (-2.70, -1.60)	−2.40 (−2.80,−2.11)	−2.11 (−2.78,−1.50)	−2.1 (−2.20,−1.50)	.202
Total hip T-Scores	−1.70 (−2.20,−1.18)	−1.63 (−2.50, −1.61)	−1.63 (−2.20,−1.00)	−1.50 (−1.90,−1.10)	.192

The significance value for the footnotes “*, †, ‡, §” is *P* < .05.

ALM = appendicular lean mass, ASMI = appendicular skeletal muscle mass index. (The same annotations apply to subsequent tables).

### 3.3. Hormone differences

The characteristics of various hormones vary significantly with sex. Compared with those in the control group, the 25 (OH)D levels in the DO group were significantly lower. Although SHBG and DHEAS seemed to be lower and FEI and FT3 seemed to be greater in the DO group, these differences were not statistically significant. Compared with those in the ND/O group, the D/NO group presented lower levels of DHEAS and FT3. However, there were no significant differences in the other indicators among the groups. As shown in Table 4.

**Table 4:** Different hormone characteristics of males in each group.

	Dynapenic obesity n = 61 (20.6%)	Dynapenic nonobesity n = 24 (8.1%)	Nondynapenic obesity n = 142 (47.9%)	Nondynapenic nonobesity n = 69 (23.3%)	*P*
Bone metabolism index					
IGF-1 (ng/mL)	126.47 (89.85, 135.50)	125.00 (103.75, 142.75)	124.00 (89.15, 140.50)§	126.47 (111.00, 165.00)‡	.077
25-hydroxyvitamin D (ng/mL)	16.70 (11.75, 19.00)§	16.05 (12.93, 19.90)	17.70 (12.80, 21.95)	19.30 (14.50, 24.10)*	.018
Parathyroid Hormone (pg/mL)	38.72 (30.10, 45.00)	32.20 (24.28, 49.88)	37.80 (29.65, 46.50)	35.50 (24.90, 40.30)	.198
Gonadal hormones					
SHBG (nmol/mL)	34.96 (30.40, 42.00)	34.96 (26.78, 45.30)	32.51 (22.20, 36.30)	34.96 (28.20, 44.00)	.014
Testosterone (ng/mL)	3.46 (2.99, 3.65)	3.47 (2.73, 4.21)	3.48 (2.75, 3.87)	3.48 (3.24, 4.38)	.152
Free androgen index	40.78 (27.59, 40.78)	40.78 (24.42, 49.22)	40.78 (33.83, 48.28)	40.78 (31.73, 45.17)	.090
Estradiol (Pg/mL)	38.15 (28.82, 46.01)	37.78 (25.06, 49.50)	38.15 (27.26, 48.00)	38.15 (29.86, 41.51)	.896
Free estrogen index	0.50 (0.28, 0.52)	0.50 (0.22, 0.66)	0.52 (0.35, 0.62)	0.43 (0.29, 0.52)	.046
FSH (mIU/mL)	13.45 (7.38, 15.90)	11.10 (7.94, 17.88)	11.36 (6.90, 14.56)	10.85 (6.99, 13.45)	.472
Luteinizing Hormone (mIU/mL)	8.37 (7.06, 9.99)	8.03 (5.50, 10.93)	7.48 (5.45, 9.38)	8.37 (6.32, 9.65)	.127
Prolactin (ng/mL)	10.40 (8.74, 12.08)	10.23 (7.31, 11.27)	10.34 (7.80, 11.83)	10.40 (7.83, 11.56)	.744
Adrenal index					
Dehydroepiandrosterone (ug/dL)	143.72 (79.80, 143.72)	100.40 (70.70, 143.72)‡	143.72 (109.25, 186.85)†	143.72 (108.70, 180.65)	.007
Cortisol (nmol/L)	391.29 (252.09, 470.76)	391.06 (316.08, 488.92)	391.29 (324.49, 465.78)	388.88 (308.23, 448.06)	.662
ACTH (Pg/mL)	46.34 (31.18, 59.67)	52.82 (38.01, 69.27)	43.88 (29.63, 56.04)	47.60 (28.31, 61.53)	.406
Thyroid index					
Free Triiodothyronine (pg/mL)	3.23 (2.83, 3.53)	2.88 (2.77, 3.21)‡	3.18 (2.91, 3.55)†	3.12 (2.90, 3.38)	.022
Free Thyroxine (ng/dL)	0.94 (0.84, 1.05)	0.95 (0.85, 1.06)	0.93 (0.83, 1.03)	0.89 (0.81, 0.99)	.222
TSH (uIU/mL)	1.64 (1.09, 2.20)	1.43 (0.95, 2.04)	1.68 (1.16, 2.33)	1.78 (1.22, 2.90)	.116
FT3/FT4	3.57 (2.78, 3.86)	3.00 (2.68, 3.53)	3.49 (3.09, 3.94)	3.48 (2.99, 4.08)	.071

The significance value for the footnotes “*, †, ‡, §” is *P* < .05.

IGF-1 = insulin-like growth factor-1, ACTH = adrenocorticotropic hormone, FSH = follicle-stimulating hormone, SHBG = sex hormone-binding globulin, ACTH = adrenocorticotropic hormone, TSH = thyroid-stimulating hormone. (The same annotations apply to subsequent tables).

For female participants, although significant differences in PTH and SHBG were observed across the 4 groups, the DO group appeared to have higher PTH and lower SHBG levels than the other groups did; however, there was no significant difference when pairwise comparisons were performed among groups (Table 5).

**Table 5:** Different hormone characteristics of females in each group.

	Dynapenic obesity n = 99 (37.9%)	Dynapenic nonobesity n = 17 (6.5%)	Nondynapenic obesity n = 123 (47.1%)	Nondynapenic nonobesity n = 22 (8.4%)	*P*
Bone metabolism index					
IGF-1 (ng/mL)	115.00 (83.60, 144.00)	116.47 (83.05, 134.50)	113.00 (83.90, 129.00)§	118.24 (116.47, 176.50)‡	.069
25-hydroxyvitamin D (ng/mL)	16.50 (12.60, 20.90)	16.20 (14.45, 20.30)	17.31 (12.50, 21.00)	16.75 (11.23, 23.83)	.975
Parathyroid Hormone (pg/mL)	41.10 (31.60, 60.30)	32.10 (23.80, 42.32)	38.60 (30.50, 46.30)	41.00 (32.25, 45.63)	.039
Gonadal hormones					
SHBG (nmol/mL)	38.50 (28.40, 51.70)	48.10 (32.90, 79.85)	38.00 (26.00, 49.50)	42.21 (36.75, 63.78)	.036
Estradiol (Pg/mL)	16.79 (15.00, 27.00)	15.00 (9.73, 26.47)	19.20 (15.00, 31.00)	23.94 (15.00, 36.00)	.385
Free estrogen index	0.19 (0.12, 0.30)	0.10 (0.07, 0.22)‡	0.22 (0.12, 0.32)†	0.24 (0.16, 0.29)	.053
Progesterone (ng/mL)	0.75 (0.54, 0.95)	0.78 (0.55, 1.39)	0.78 (0.45, 0.94)	0.78 (0.38, 0.90)	.804
Prolactin (ng/mL)	12.09 (9.18, 14.51)	13.36 (8.74, 14.97)	11.23 (8.66, 13.56)	12.26 (7.08, 12.64)	.330
Luteinizing Hormone (mIU/mL)	28.85 (20.48, 39.98)	33.08 (15.72, 45.35)	27.85 (18.54, 37.91)	54.22 (21.68, 173.15)	.294
FSH (mIU/mL)	60.26 (44.53, 80.15)	48.84 (35.02, 68.08)	60.35 (40.65, 74.38)	60.35 (41.87, 72.39)	.444
Testosterone (ng/mL)	0.32 (0.19, 0.42)	0.26 (0.11, 0.53)	0.32 (0.22, 0.41)	0.30 (0.19, 0.36)	.976
Free androgen index	3.24 (1.66, 4.72)	2.06 (1.01, 4.03)	2.98 (1.74, 4.68)	3.93 (1.36, 4.03)	.567
Adrenal index					
Dehydroepiandrosterone (ug/dL)	70.10 (42.80, 94.20)	64.90 (42.50, 83.74)	83.27 (50.80, 109.90)	83.27 (57.10, 143.20)	.060
Cortisol (nmol/L)	380.39 (280.04, 480.44)	393.24 (380.39, 474.27)	359.57 (254.12, 447.34)	380.39 (295.63, 532.76)	.049
ACTH (Pg/mL)	38.45 (25.93, 49.01)	37.44 (24.89, 46.85)	36.57 (24.21, 46.54)	40.12 (23.44, 62.55)	.851
Thyroid index					
Free Triiodothyronine (pg/mL)	2.99 (2.74, 3.30)	3.02 (2.76, 3.28)	2.95 (2.67, 3.31)§	3.39 (2.79, 3.74)‡	.068
Free Thyroxine (ng/dL)	0.94 (0.82, 1.05)	0.99 (0.85, 1.08)	0.93 (0.81, 0.99)	0.99 (0.86, 1.10)	.223
TSH (uIU/mL)	1.99 (1.31, 2.85)	1.75 (1.61, 2.13)	1.99 (1.35, 3.01)	2.08 (1.59, 3.03)	.782
FT3/FT4	3.24 (2.73, 3.76)	3.05 (2.46, 3.68)	3.28 (2.86, 3.64)	3.29 (3.01, 3.95)	.551

The significance value for the footnotes “†, ‡, §” is *P* < .05.

### 3.4. Ordinal logistic regression

Finally, to determine the relationships between changes in muscle strength/fat mass and hormone levels in different sexes, a multiple OLR model was further established (Table 6). To ensure the robustness of the analysis, Model 1 included hormone types that showed differences in the baseline analysis (*P* < .1).

**Table 6:** The relationship between changes in muscle strength/fat mass and hormone levels in different sexes.

	Model-1				Model-2				Model-3			
	β	Wald	*P*	OR (95%CI)	β	Wald	*P*	OR (95%CI)	β	Wald	*P*	OR (95%CI)
**Female**												
DHEAS (ug/dL)	−0.005	3.894	.048	0.99 (0.99, 1.00)	−0.005	4.745	0.029	0.99 (0.99, 1.00)	−0.005	4.747	0.029	0.99 (0.99, 1.00)
PTH (pg/mL)	0.016	7.926	.005	1.02 (1.01, 1.03)	0.015	7.538	0.006	1.02 (1.01, 1.03)	0.013	5.836	0.016	1.01 (1.00, 1.02)
Male												
25 (OH)D (ng/mL)	−0.02	4.203	.040	0.98 (0.96, 1.00)	−0.02	4.02	0.045	0.98 (0.96, 1.00)	-0.023	4.00	0.045	0.98 (0.96, 1.00)
IGF-1 (ng/ml)	−0.005	5.712	.017	0.99 (0.99, 1.00)								

Model-1:unadjusted.

Model-2:adjusted for age, drink, and smoke.

Model-3:adjusted for age, drink, smoke, HbA1c, and DCSI score.

In the end, among male participants, the same result was obtained in both the unadjusted and adjusted models; that is, as muscle strength decreased and fat mass increased, 25 (OH)D showed a negative correlation. In addition, we also found that although the DO group had lower IGF-1 levels than the control group did in Model 1, the difference was no longer statistically significant after adjusting for demographic characteristics and diabetes-related parameters.

Different results were obtained among female participants; as muscle strength and fat mass changed, DHEAS showed a negative correlation, whereas PTH showed a positive correlation. These results were statistically significant in all 3 models.

## 4. Discussion

This study confirms for the first time that DO is prevalent in patients of both sexes with T2DM (20.6% of males and 37.9% of females), and such a population is more likely to be advanced in age, have slower mobility, have reduced muscle mass, have poorer nutritional status, and have a greater low-grade inflammatory tendency than their corresponding controls. More importantly, beyond these shared characteristics, our analyses further revealed that DO patients display a distinct sex-specific hormonal phenotypic pattern: specifically, in males, decreased serum levels of 25 (OH)D is significantly associated with an increased risk of DO even after adjusting for multiple confounding factors, whereas in females, the condition tends to be independently strongly associated with decreased serum DHEAS or elevated PTH levels.

Earlier data have demonstrated a robust association between low serum 25 (OH)D levels and the development of sarcopenia, obesity and SO.^[12,20–22]^ All these findings support our results, which revealed an inverse correlation between 25 (OH)D levels and decreasing muscle strength along with increasing fat mass in males. However, the results concerning the therapeutic efficacy of vitamin D supplementation in SO populations are quite inconsistent.^[23,24]^ The differences in results may be related to variations in the baseline concentrations of 25 (OH)D, forms and doses of supplementation, and differing definitions of SO across studies. Therefore, further research is needed to determine whether vitamin D3 supplementation is warranted for DO populations. In contrast, the pleiotropic effects of vitamin D on both skeletal muscle and adipose tissue have been validated in multidimensional studies in recent decades. On the one hand, available evidence suggests that vitamin D may regulate muscle mitochondrial function through vitamin D receptor-mediated gene expression.^[25]^ This genomic pathway may also modulate body composition by influencing muscular calcium transport and phospholipid metabolism.^[26,27]^ Furthermore, vitamin D directly regulates the diameter and quantity of type II (fast-twitch) muscle fibers.^[28]^ On the other hand, vitamin D deficiency may compromise immune function, reduce antioxidant and anti-inflammatory capacity, and affect adipogenesis, lipolysis, and adipose tissue inflammation.^[29]^

Our research also revealed higher FT3 levels in male DO patients. This result aligns with prior research.^[30–32]^ Further subgroup analyses of the overweight and obese populations in this study revealed that the group with the highest fat content and lowest muscle composition presented even higher FT3 levels than did the group with the lowest fat content and highest muscle composition.^[33]^ However, the clinical implications of elevated FT3 in SO patients are unclear. A compensatory or adaptive mechanism may be involved, since potential drivers include adipose tissue expansion driving hyperleptinemia, which secondarily modulates thyroid axis activity,^[34]^ and upregulated DIO1 expression in obese individuals, accelerating FT3 generation from FT4 and thus modifying metabolic expenditure.^[35]^ We also observed significantly lower IGF-1 levels in the male DO group before adjustment for confounding factors; however, this difference lost statistical significance after multivariate correction, suggesting that age and diabetes-related characteristics may influence IGF-1 concentrations.

Interestingly, the study revealed other modifiable hormones that are also closely related to the progression of DO; that is, baseline DHEAS levels were lower in male DO patients than in controls. Regression analysis also confirmed significantly reduced DHEAS in females, with a trend toward decreased muscle strength and increased fat mass. Prior evidence has also demonstrated that declining DHEAS levels not only correlate with elevated adiposity but also contribute to the loss of muscle mass and function,^[36,37]^ and the underlying molecular drivers governing this phenomenon have also been confirmed: as a T precursor, DHEAS promotes anabolic effects, stimulates IGF-1 production and alleviates oxidative stress in skeletal muscle.^[38,39]^ The adipogenic effects of DHEAS seem to be similarly well established and are mediated through increased tyrosine phosphorylation of insulin receptor substrate 1/insulin receptor substrate 2 and stimulation of phosphoinositide 3-kinase activity in adipocytes.^[40,41]^ However, numerous studies have indicated that DHEAS supplementation fails to improve body composition or physical function,^[42–45]^ although not all are the same.^[46,47]^ Marked variations in sample size, baseline characteristics of the study populations, follow-up duration, and DHEA dosage across studies may account for the discrepancy. Furthermore, our findings also indicate that elevated PTH levels are closely associated with increasing adiposity alongside decreased muscle strength.^[48–50]^ Moreover, we also observed lower baseline calcium levels in the DO group than in the control group. Previous studies have demonstrated that vitamin D supplementation further reduces PTH concentrations.^[51,52]^ These findings suggest that supplementation with vitamin D and calcium may mitigate the risk of DO, although large-scale longitudinal studies are warranted for validation.

Our study has several strengths. For example, prior research on hormonal characteristics in patients with SO has focused mainly on single hormonal axes. Moreover, few studies have been conducted among individuals with multiple metabolic disorders. This study also has limitations. First, the cross-sectional design precludes causal inferences. Second, we measured sex hormones using chemiluminescence assays rather than the gold-standard liquid chromatography–tandem mass spectrometry (LC–MS/MS) method. It is well established that different assay platforms can yield substantially different results for steroid hormones, particularly for sex hormones. Third, the cross‑sectional nature of this study precludes us from controlling for all unmeasured confounders. Future large‑scale prospective interventional investigations are thus necessary to verify the observed associations. Furthermore, this study was conducted at a single center in China, and our findings require further validation in multi‑ethnic and multicenter studies.

## 5. Conclusion

In summary, DO is more common in middle-aged and older adults of both sexes with T2DM, which is potentially linked to suboptimal nutritional status, reduced physical activity, and low-grade inflammation overexposure. More importantly, our study also identified several modifiable hormones whose circulating levels are closely associated with DO. Nevertheless, whether interventions targeting these hormones can improve body composition or function and prognosis in DO patients with T2DM still requires rigorous investigation.

## Acknowledgments

We thank all the patients and their families for participating in the study.

## Author contributions

**Formal analysis:** Di Yu, Qin Ou.

**Investigation:** Di Yu, Qin Ou, Bo Xie, Yinghao Li.

**Methodology:** Di Yu, Qin Ou.

**Resources:** Kun Liao, Rong Li, Mei Mei.

**Conceptualization:** Bo Zhou.

**Project administration:** Bo Zhou.

**Supervision:** Bo Zhou.

**Writing – original draft:** Di Yu, Qin Ou.

**Writing – review & editing:** Di Yu, Qin Ou.
